# Synergistic activity of everolimus and 5‐aza‐2′‐deoxycytidine in medullary thyroid carcinoma cell lines

**DOI:** 10.1002/1878-0261.12070

**Published:** 2017-06-21

**Authors:** Giovanni Vitale, Alessandra Dicitore, Daniele Pepe, Davide Gentilini, Elisa S. Grassi, Maria O. Borghi, Giulia Gelmini, Maria C. Cantone, Germano Gaudenzi, Gabriella Misso, Anna M. Di Blasio, Leo J. Hofland, Michele Caraglia, Luca Persani

**Affiliations:** ^1^ Department of Clinical Sciences and Community Health (DISCCO) University of Milan Italy; ^2^ Laboratory of Endocrine and Metabolic Research Istituto Auxologico Italiano IRCCS Milan Italy; ^3^ I‐BioStat Hasselt University Belgium; ^4^ Molecular Biology Laboratory Istituto Auxologico Italiano Milan Italy; ^5^ Experimental Laboratory of Immuno‐rheumatologic Researches Istituto Auxologico Italiano IRCCS Milan Italy; ^6^ Department of Biochemistry, Biophysics and General Pathology Second University of Naples Italy; ^7^ Section Endocrinology Department of Internal Medicine Erasmus Medical Center Rotterdam The Netherlands

**Keywords:** 5‐aza‐2′‐deoxycytidine, everolimus, medullary thyroid cancer, mTOR, neurotrophin pathway, NGFR

## Abstract

Medullary thyroid cancer (MTC) is a tumor highly resistant to chemo‐ and radiotherapy. Drug resistance can be induced by epigenetic changes such as aberrant DNA methylation. To overcome drug resistance, we explored a promising approach based on the use of 5‐aza‐2′‐deoxycytidine (AZA), a demethylating agent, in combination with the mTOR inhibitor everolimus in MTC cells (MZ‐CRC‐1 and TT). This combined treatment showed a strong synergistic antiproliferative activity through the induction of apoptosis. The effect of everolimus and/or AZA on genome‐wide expression profiling was evaluated by Illumina BeadChip in MZ‐CRC‐1 cells. An innovative bioinformatic pipeline identified four potential molecular pathways implicated in the synergy between AZA and everolimus: PI3K‐Akt signaling, the neurotrophin pathway, ECM/receptor interaction, and focal adhesion. Among these, the neurotrophin signaling pathway was most directly involved in apoptosis, through the overexpression of NGFR and Bax genes. The increased expression of genes involved in the NGFR‐MAPK10‐TP53‐Bax/Bcl2 pathway during incubation with AZA plus everolimus was validated by western blotting in MZ‐CRC‐1 cells. Interestingly, addition of a neutralizing anti‐NGFR antibody inhibited the synergistic cytotoxic activity between AZA and everolimus. These results open a new therapeutic scenario for MTC and potentially other neuroendocrine tumors, where therapy with mTOR inhibitors is currently approved.

**Abbreviations**
AZA: 5‐Aza‐2′‐deoxycytidine
BDNF: brain‐derived neurotrophic factor
CI: combination index
DEGs: differentially expressed genes
DRI: dose reduction index
FDR: false discovery rate
MTC: medullary thyroid cancer
NGF: nerve growth factor
NT‐3: neurotrophins‐3
NT‐4/5: neurotrophins‐4/5
NT‐6: neurotrophins‐6
PARP: poly(ADP‐ribose) polymerase
PF: potentiation factor
PI: propidium iodide
SAM: significance analysis of microarray
SEM: structural equation modeling
SPIA: signaling pathway impact analysis

## 1 Introduction

Medullary thyroid cancer (MTC) is a neuroendocrine tumor originating from parafollicular C cells, and it is highly resistant to chemo‐ and radiotherapy (Vitale *et al*., 2001). The hyperactivation of the PI3K/Akt/mTOR cascade has a relevant role in the pathogenesis and progression of MTC. In fact, most of the pro‐oncogenic effects of RET and RAS mutations are modulated by the activation of PI3K/Akt/mTOR pathway (Lyra *et al*., 2014; Manfredi *et al*., 2015). Few preliminary studies showed promising antitumor effects of mTOR inhibitors, such as everolimus, in MTC (Druce *et al*., 2012; Faggiano *et al*., 2012; Heilmann *et al*., 2016; Lim *et al*., 2013). A recent phase II study, assessing efficacy and tolerability of everolimus on tumor progression in patients with advanced thyroid cancer, showed stable disease in five (71%) of seven patients with MTC and a low toxicity profile for everolimus (Schneider *et al*., 2015).

Although everolimus has been reported to inhibit cell proliferation and angiogenesis in several human tumors, long‐term treatment with mTOR inhibitors can be frustrated by the induction of resistance. Moreover, the anticancer effect of everolimus may be limited because of the intrinsic lack of inhibition on mTORC2 and the activation of survival pathways in cancer cells (Chan and Kulke, 2014). On this basis, anticancer treatment with everolimus as monotherapy may not be optimal, supporting the rationale for a combined treatment approach with other targeted agents.

Epigenetic alterations, including dysregulated protein acetylation and DNA methylation, affect gene expression and contribute to tumorigenesis in several tumors (Hervouet *et al*., 2013; Walenkamp *et al*., 2014), including MTC (Vitale *et al*., 2016). PI3K/Akt/mTOR pathway is frequently deregulated in several malignancies through epigenetic alterations (Goel *et al*., 2004). Epigenetic alterations appear to be also implicated in the development of resistance to mTOR inhibitors (Bihani *et al*., 2015; Juengel *et al*., 2012). In fact, histone deacetylase inhibitors are able to resensitize renal carcinoma cells to mTOR inhibitors (Bihani *et al*., 2015; Juengel *et al*., 2012, 2014). However, most of the epigenetic mechanisms of resistance to mTOR inhibitors, particularly those concerning aberrant DNA methylation, remain poorly defined.

In this article, we have explored a novel approach to potentiate the antitumor activity of everolimus, based on the use of 5‐aza‐2′‐deoxycytidine (AZA), a well‐established demethylating agent, in MTC.

## 2 Materials and methods

### 2.1 Cell lines and reagents

Everolimus and AZA were kindly supplied by Novartis Pharma (Basel, Switzerland) and Sigma‐Aldrich (St. Louis, MO, USA), respectively. Both drugs were dissolved in DMSO and stored at −20 °C. Human MTC cell lines (MZ‐CRC‐1 and TT) were provided from Prof. Lips (Utrecht, the Netherlands). Both cell lines were cultured at 37 °C and 5% CO_2_ in F‐12 with Kaighn's modification medium supplemented with 10% fetal bovine serum, 2 nm glutamine, and 10^5^ U·L^−1^ penicillin/streptomycin. HEK‐293 (ATCC) human embryonic kidney cell line was cultured at 37 °C and 5% CO_2_ in DMEM high glucose medium, without sodium pyruvate, supplemented with 10% fetal bovine serum, 2 nm glutamine, and 10^5^ U·L^−1^ penicillin/streptomycin.

### 2.2 Cell viability assessment

MTC cells were plated in 96‐well plates (3 × 10^4^ cells per well) and treated with various concentrations of compounds (range 10^−1^–10^2 ^nm for everolimus, 10^−3^–10^5 ^nm for AZA). Cells growing in drug‐free medium and vehicle‐treated were used as control. After 3 days, medium and drugs were replaced. After 6 days, viability of cells was measured by the MTT assay (3‐(4,5‐dimethylthiazol‐2‐yl)‐2.5‐diphenyltetrazolium), as previously described (Vitale *et al*., 2012). Under the same conditions, the *in vitro* toxicity of everolimus and AZA at synergistic concentrations was examined through MTT assay in HEK‐293 cells.

### 2.3 Drug combination studies

MTC cells were seeded in 96‐well plates with medium without and with everolimus and/or AZA at different concentrations. After 3 days, medium and compounds were refreshed. Cell viability was evaluated after 6 days of treatment using MTT assay. Three combinations were tested for each schedule: equiactive doses of the two agents (IC_50_), higher relative doses of everolimus (IC_75_ of everolimus/IC_25_ of AZA), and higher relative doses of AZA (IC_25_ of everolimus/IC_75_ of AZA). Assessment of synergistic interaction between drugs was made with calcusyn software (Biosoft, Ferguson, MO, USA). Combination index (CI) values of < 1, 1, and > 1 are suggestive of synergy, additivity, and antagonism, respectively (Chou and Talalay, 1984; Chou *et al*., 1994). We also evaluated the dose reduction index (DRI), providing the magnitude of how much the dose of each drug in a synergistic combination may be reduced at a given effect level compared with the doses of each drug alone; the potentiation factor, defined as the ratio of the IC_50_ of either everolimus or AZA alone to the IC_50_ of everolimus and AZA in combination.

### 2.4 Cell cycle analysis

Cells were plated in six‐well plates (3 × 10^5^ cells/well) in duplicate with medium without (control group) and with everolimus and/or AZA. After 3 days, medium and drugs were refreshed. After 6 days, cells were collected, stained with propidium iodide (PI) (Sigma‐Aldrich), and analyzed using a FACScalibur flow cytometer (Becton Dickinson, Erembodegem, Belgium) and cellquest pro Software, as previously described (Vitale *et al*., 2007).

### 2.5 Flow cytometric analysis of apoptosis

Cells were plated in six‐well plates and incubated with everolimus and/or AZA, as reported in the section of cell cycle analysis. After 6 days of treatment, cells were collected and stained with Annexin V‐FITC (BD Pharmingen, San Diego, CA, USA) and PI, and all samples were analyzed with FACScalibur, as previously reported (Vitale *et al*., 2006).

### 2.6 Western blot

MZ‐CRC‐1 cells were plated in six‐well plates and incubated with everolimus and/or AZA, as previously reported. After 6 days of treatment, cells were scraped, washed in PBS, and resuspended in RIPA lysis buffer. Lysates were centrifuged, and protein analysis was conducted on supernatant. Cell extracts (50 μg per lane) were separated on NuPage 4–12% Bis/Tris Gels and transferred with iBlot System (Life Technologies, Carlsbad, CA, USA). Membranes were blocked with 5% milk TBS‐T and incubated with specific antibodies overnight at 4 °C: anticaspase‐3 and poly(ADP‐ribose) polymerase (PARP), anticleaved caspase‐3 and PARP, anti‐Bcl2, anti‐Bax, anti‐mTOR, anti‐phospho‐mTOR (Ser2448), anti‐4E‐BP1, anti‐phospho‐4E‐BP1 (Ser 65), anti‐MAPK10, anti‐phospho‐MAPK8/9/10 (Thr183/Tyr185), anti‐phospho‐p53 (Ser15) (Cell Signaling Technology, Beverly, MA, USA), and anti‐NGFR (Sigma‐Aldrich). Blots were detected by the Luminata Forte ECL Kit (Millipore, Darmstadt, Germany) after incubation with HRP‐conjugated mouse and rabbit secondary antibodies (dilution 1 : 1000 for anti‐MAPK10, anti‐phospho‐MAPK8/9/10 and anti‐phospho‐p53; 1 : 5000 for anticaspase‐3; and 1 : 10 000 for other antibodies) and then exposed to X‐ray film. Bands of interest were quantified through imagej software version 1.47 (Wayne Rasband, NIH, Bethesda, MA, USA). Results were normalized against the level of actin expression in each sample, and the intensities of the bands were expressed as arbitrary units when compared to those of the untreated cells.

### 2.7 Gene expression profiling

MZ‐CRC‐1 cells were seeded in six‐well plates and treated with everolimus and/or AZA, as previously reported. After 6 days, total RNA was extracted using Trizol Reagent (Life Technologies) and purified by RNeasy Mini Kit (Qiagen, Hilden, Germany). Amplification‐grade DNase I (Life Technologies) was used to eliminate residual genomic DNA from RNA samples. Samples with an A260/A280 ratio falling in the range 1.8–2.1 were used for experiments.

The Illumina TotalPrep RNA Amplification Kit (Ambion, Foster City, USA) was employed using 200 ng of total RNA as starting material. Labeled cRNA (750 ng) was hybridized to human HT‐12 v3 BeadChip arrays (Illumina, San Diego, CA, USA) according to the manufacturer's recommendation. Fluorescent images were obtained with a BeadArray reader and processed with the beadscan software (Illumina, San Diego, CA, USA).

### 2.8 Microarray gene expression analysis

Significance analysis of microarray was employed to identify differentially expressed genes (DEGs) by comparing the expression value of each gene between every group of treatment *vs* the untreated control, as previously described (Tusher *et al*., 2001). All the delta values were selected in order to provide a false discovery rate < 0.05. Once determined the DEGs, the next step was to discover which biological pathways were associated with the list of DEGs through signaling pathway impact analysis (SPIA) (Tarca *et al*., 2009), an algorithm of third generation that takes in consideration not only the identity of the genes but also the topology of the pathway. These aspects were reported by the probability values: pPERT (reflecting the amount of perturbation measured in each pathway) and pNDE (probability of obtaining a number of DEGs on the given pathway at least as large as the observed one). These two types of evidence, pPERT and pNDE, were finally combined into one global probability value, pGFdr, which was used to rank the pathways and to test the research hypothesis that the pathway was significantly involved in the condition under the study. To refine the SPIA, a downstream analysis based on the structural equation modeling (SEM) was performed (Pepe and Do, 2015; Pepe and Grassi, 2014). The method consists in the understanding how the DEGs, source of perturbation, propagated the perturbation in the biological network composed by the significant pathways. The module was obtained by the detection and fusion in a unique model of all directed shortest paths that put in communication the DEGs present in the significant pathways. This allowed (a) to overcome the limits of the classical pathway analysis that considers the pathways as separate entities and (b) to detect the biological network where the drug modules are searched.

### 2.9 DNA preparation and infinium methylation 450K array

MZ‐CRC‐1 cells were plated in six‐well plates and treated with everolimus and/or AZA, as previously reported. After 6 days, total DNA was extracted using the DNeasy Blood & Tissue Kit (Qiagen). Quality control and quantification of DNA were performed before and after bisulfite conversion. DNA was quantified with NanoDrop (NanoDrop Products Thermo Scientific, Wilmington, DE, USA) and by fluorometric reading (Quant‐iT™ PicoGreen^®^ dsDNA Assay Kit); quality was assessed by visualization of genomic DNA on 1% agarose gel electrophoresis. Only nonfragmented DNA samples with a concentration higher than 50 ng·μL^−1^ were subsequently processed. The genomic DNA was treated with sodium bisulfite using the EZ DNA Methylation Kit™ (Zymo Research, Irvine, CA, USA); the technique requires only 500 ng of input DNA. Four microliters of bisulfite‐converted DNA were used for hybridization on Infinium HumanMethylation 450K BeadChip, according to Illumina's standard protocol. Data were acquired through Illumina HiScan SQ scanner. Image intensities were extracted using genomestudio software v2010.3 (Illumina). The methylation score for each CpG site was represented as β‐values according to the fluorescent intensity ratio between methylated and unmethylated probes. β‐Values may range between 0 (completely unmethylated) and 1 (completely methylated). Illumina Methylation 450K raw data were analyzed using the rnbeads analysis software package (Assenov *et al*., 2014). Sites overlapping SNPs were firstly removed from the analysis as well as probes on sex chromosomes. Probes and samples of highest impurity were removed from the dataset using the Greedycut algorithm. We have considered every β‐value to be unreliable when its corresponding detection *P*‐value was not below the threshold (*T* = 0.05). The background was subtracted using the methylumi package (method ‘noob’). The signal intensity values were normalized using the SWAN normalization method, as implemented in the minfi package (Aryee *et al*., 2014). The ‘Shapiro.test’ function provided in the R package ‘stats’ was applied to test normality among variables. The ‘kruskal.test’ function provided in the R package ‘stats’ was used to test differences among treated and untreated groups for all nonparametric data. DunnTest provided in the R package ‘dunn.test’ has been applied as *post hoc* test.

### 2.10 Statistical analysis

All experiments were performed at least three times. Statistical differences among groups were first evaluated by the ANOVA test, followed by *post hoc* test (Newman–Keuls). A *P* value < 0.05 was considered significant. The values reported in the figures represent the mean ± standard error of the mean. For statistical analysis, graphpad prism 5.0 was used (GraphPad Software Inc., La Jolla, CA, USA).

## 3 Results

### 3.1 Pharmacological combination between everolimus and AZA on cell proliferation

**Figure 1:**
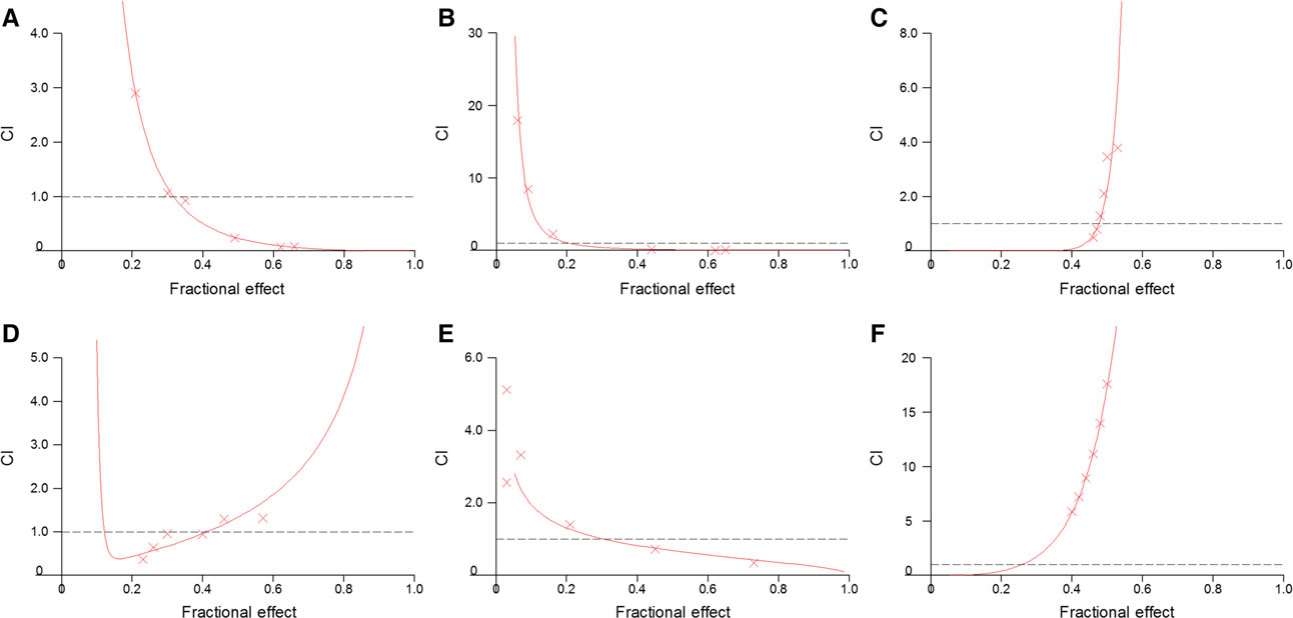
Evaluation of synergism between everolimus (EV) and 5‐aza‐2′‐deoxycytidine (AZA) in medullary thyroid cancer cells by isobologram analysis. These experiments were performed with MTT assay. Combination index (CI)/fractional effect curves were elaborated with the dedicated software calcusyn (developed by Chou and Talalay) as described in Materials and methods. Curves show the CI *vs* the fraction of medullary thyroid carcinoma cells MZ‐CRC‐1 (A–C) and TT (D–F) affected by the EV/AZA combinations at 50 : 50 (A/D), 25 : 75 (B/E), 75 : 25 (C/F) cytotoxic ratios. CI represents the assessment of synergy induced by drug interaction. In detail, CI values of < 1, 1, and > 1 indicate synergy, additivity, and antagonism, respectively. Each point (represented in graph by x‐mark) is the mean of at least four different replicates. The statistical significance of each point was evaluated with ANOVA, and the derived *P* values were always less than 0.01.

In MZ‐CRC‐1 cells, the combination of everolimus and AZA was highly synergistic when either of the two drugs was used with lower concentrations of everolimus (IC_25_ everolimus:IC_75_ AZA) or with equitoxic concentrations (IC_50_ everolimus:IC_50_ AZA) (Table 1, Fig. 1). The DRI_50_ (DRI calculated for 50% cell survival) was 8.8 for everolimus and 101 for AZA (when drugs were used at IC_25_ everolimus:IC_75_ AZA) and 4.3 for everolimus and 590 for AZA (at equitoxic concentrations) (Table 1).

**Table 1:** Combination index (CI), dose reduction index (DRI), and potentiation factor (PF), according to the different cytotoxic ratio of everolimus (EV) and 5‐aza‐2′‐deoxycytidine (AZA) combination in MZ‐CRC‐1 and TT cell lines after 6 days of treatment

Cell line	Cytotoxic ratio	ED_50_ (nm)		CI_50_ a	DRI_50_ b		PF^c^		Interpretation
		EV	AZA		EV	AZA	EV	AZA	
MZ‐CRC‐1	EV‐AZA (50 : 50)	12.4	35.6	0.2	4.3	5.9 × 10^2^	1.8	1.1 × 10^3^	Strong synergism
	EV‐AZA (25–75)	2.1	1.4 × 10^2^	0.1	8.8	1.0 × 10^2^	10.4	2.8 × 10^2^	Strong synergism
	EV‐AZA (75–25)	1.3 × 10^2^	3.2 × 10^−1^	2.3	−	−	−	−	Antagonism
TT	EV‐AZA (50 : 50)	9.2	3.4 × 10^−1^	1.3	−	−	−	−	Antagonism
	EV‐AZA (25–75)	1.2	8.0	0.7	1.5	6.4 × 10^2^	6.1	11	Synergism
	EV‐AZA (75–25)	91.1	1.2 × 10^−3^	17	−	−	−	−	Antagonism

a CI_50_ was calculated for 50% cell survival (ED_50_) by isobologram analyses performed with calcusyn software.

b DRI was calculated in case of synergism. It represents the order of magnitude (fold) of dose reduction obtained for ED_50_ effect in combination setting as compared to each drug alone.

c PF was calculated in case of synergism as the ratio between the IC_50_ of either everolimus or AZA alone and the IC_50_ of everolimus or AZA in combination setting.

In TT cells, a synergistic activity was similarly detected when lower concentrations of everolimus were adopted (IC_25_ everolimus:IC_75_ AZA), while antagonistic effects were observed with relatively higher concentrations of everolimus (IC_75_ everolimus:IC_25_ AZA) (Table 1, Fig. 1).

The optimal results (lowest CI values) were obtained when the two drugs were used at lower doses of everolimus in MZ‐CRC‐1 cells. Therefore, we have performed subsequent experiments using 2.1 nm (IC_25_) of everolimus and/or 1.4 × 10^2^ nm (IC_75_) of AZA in MZ‐CRC‐1 cells.

### 3.2 *In vitro* toxicity assessment

**Figure 2:**
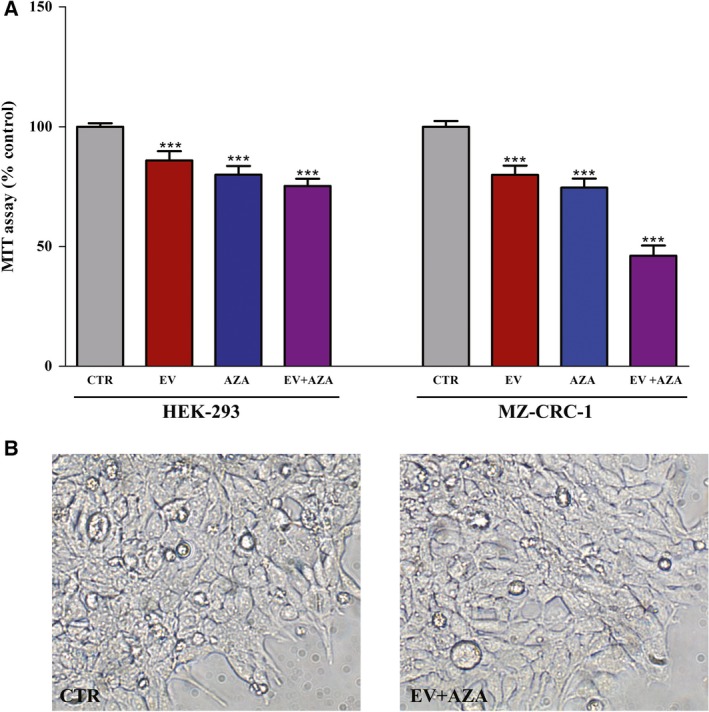
*In vitro* toxicity profile of everolimus (EV) and 5‐aza‐2′‐deoxycytidine (AZA). Cells were treated for 6 days with EV (2.1 nm) and/or AZA (1.4 × 10^2^ nm). (A) The cytotoxicity was measured by MTT cell viability assay in HEK‐293 and MZ‐CRC‐1 cell lines. (B) The morphology of HEK‐293 cells was determined after 6 days of exposure to drug‐free medium (CTR) and EV plus AZA. Images were captured using a phase‐contrast microscopy at × 20 magnification. ***P < 0.001.

The effect of drug combination on the cell viability of HEK‐293 cell line, derived from human embryonic kidney cells, was evaluated through MTT assay to roughly predict the toxicity profile of both everolimus and AZA in a noncancer cell model. Combined treatment with everolimus and AZA, at concentrations showing synergistic antiproliferative activity in MZ‐CRC‐1 cells, inhibited viability of HEK‐293 cells (−25%) to a lesser extent than that detected in MTC cells (−54%) (Fig. 2A). Moreover, we did not observe any change in morphology of HEK‐293 cells after treatment with everolimus and AZA compared to untreated control (Fig. 2B).

### 3.3 Effects of everolimus and AZA on cell cycle

**Figure 3:**
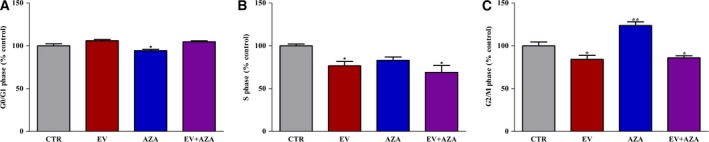
Cell cycle distribution, detected by FACS analysis, in propidium iodide‐stained MZ‐CRC‐1 cells after 6 days of treatment with 2.1 nm everolimus (EV) and/or 1.4 × 10^2^ nm 5‐aza‐2′‐deoxycytidine (AZA). Control values have been set to 100%. *P < 0.05 **P < 0.01.

AZA alone slightly, but significantly decreased cell population in G_0_/G_1_ phase (−6% *vs* untreated control, *P* < 0.05) and increased cell number in G_2_/M phase (+23% *vs* untreated control, *P* < 0.01), suggesting a cell cycle arrest in G_2_/M phase (Fig. 3). Everolimus alone significantly decreased cells percentage in both S (−24% *vs* untreated control, *P* < 0.05) and G_2_/M phase (−15% *vs* untreated control, *P* < 0.05). This effect was comparable to that observed during the synergistic combination of everolimus plus AZA (S phase: −27% *vs* untreated control, G_2_/M phase: −14% *vs* untreated control, both *P* < 0.05, Fig. 3).

### 3.4 Effects of everolimus and AZA on apoptosis

**Figure 4:**
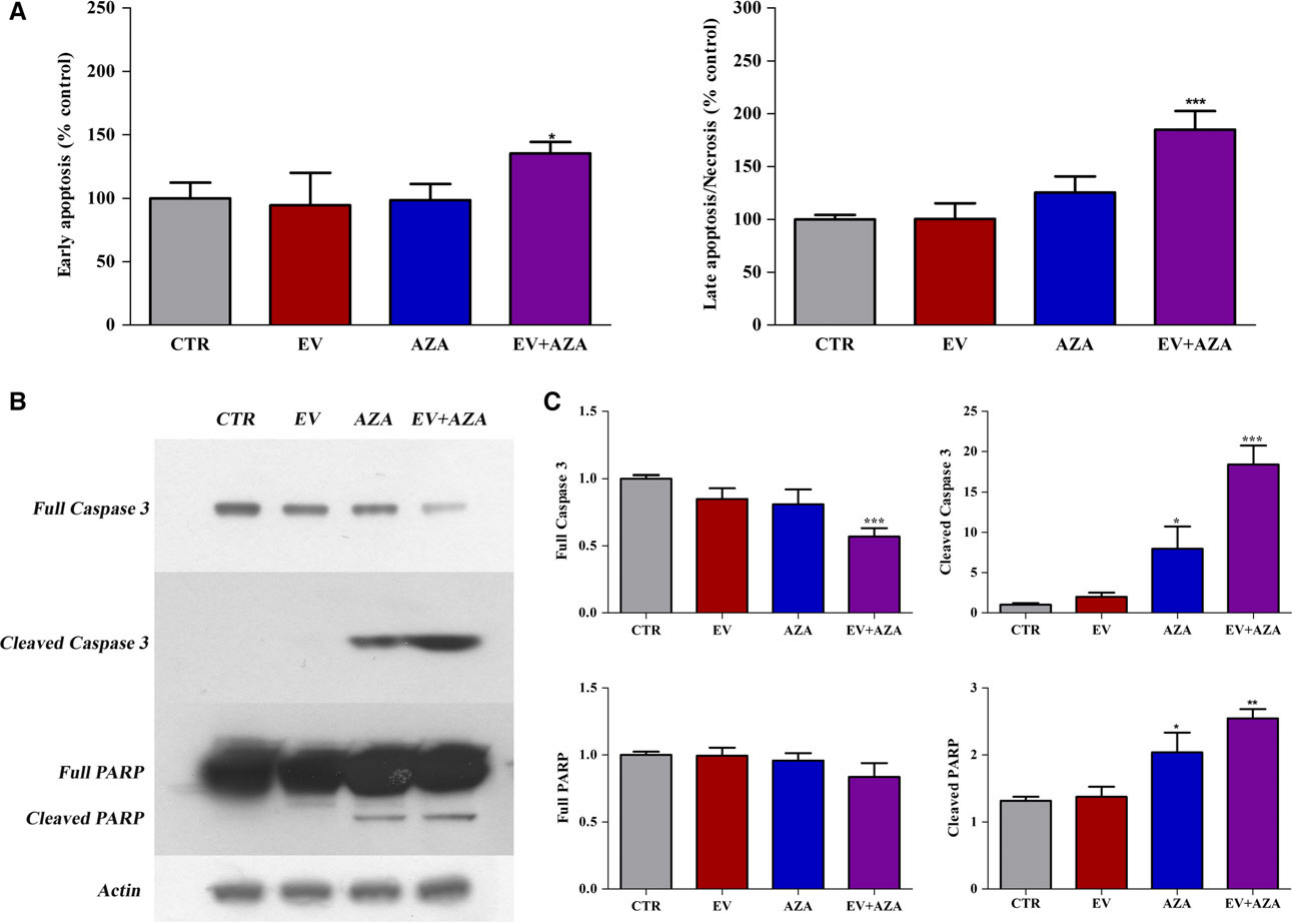
Effects of 2.1 nm everolimus (EV) and/or 1.4 × 10^2^ nm 5‐aza‐2′‐deoxycytidine (AZA) on apoptosis in MZ‐CRC‐1 cells after 6 days of incubation. (A) Flow cytometry with Annexin V and propidium iodide. Values of early apoptotic cells and late apoptotic/necrotic cells were expressed as percentage compared with the untreated control. (B) Representative western blot analysis of apoptotic markers (full and cleaved forms of caspase 3 and PARP). Actin was used as a loading control. (C) Quantification of western blot analysis from at least three independent experiments. *P < 0.05; **P < 0.01; ***P < 0.001.

Everolimus did not change the percentages of early apoptotic and late apoptotic/necrotic cells compared with the untreated control. AZA induced a statistically nonsignificant increase in the population of late apoptotic/necrotic cells. Interestingly, the AZA/everolimus combination significantly increased the percentage of early apoptotic (+35% *vs* control, *P* < 0.05) and late apoptotic/necrotic (+90% *vs* control, *P* < 0.001) cells (Fig. 4A).

Western blot analysis of key executioners of apoptosis, such as caspase‐3 and PARP, further confirmed these data (Fig. 4B,C). After 6 days of treatment, the AZA/everolimus combination significantly increased caspase‐3 degradation, resulting in increased expression of cleaved caspase‐3 and decreased expression of full caspase‐3. A moderate increase in cleaved caspase‐3 has been detected after AZA alone. Similarly, the activity of PARP was moderately stimulated after 6 days of treatment with AZA, and its combination with everolimus enhanced again this effect. No significant changes in caspase‐3 activity and PARP cleavage were detected after the incubation of MZ‐CRC‐1 cells with everolimus alone.

### 3.5 Effects of everolimus and AZA on mTOR activation

**Figure 5:**
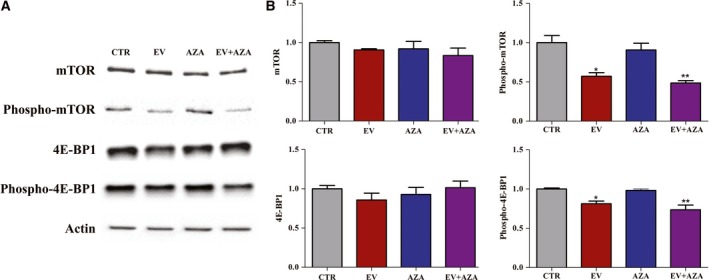
Effects of 2.1 nm everolimus (EV) and/or 1.4 × 10^2^ nm 5‐aza‐2′‐deoxycytidine (AZA) on protein expression of total and phosphorylated mTOR and 4E‐BP1 in MZ‐CRC‐1 cells after 6 days of incubation. (A) Representative western blot analysis. Actin was used as a loading control. (B) Quantification of western blot analysis from at least three independent experiments. *P < 0.05; **P < 0.01.

No detectable impact on total mTOR and 4E‐BP1 protein levels has been observed during incubation with everolimus and/or AZA in MZ‐CRC‐1 cells (Fig. 5). Everolimus alone significantly reduced both mTOR and 4E‐BP1 phosphorylation. A similar effect has been observed after everolimus in combination with AZA, while AZA alone did not significantly affect this process (Fig. 5).

### 3.6 Gene expression analysis

To clarify the molecular bases for synergy between these two compounds, gene expression profiles with or without everolimus and/or AZA treatment were evaluated in MZ‐CRC‐1 cells. Following treatment with everolimus or AZA alone, 72 DEGs (Table S1) and 16 DEGS (Table S2) were identified, respectively. On the other hand, 74 DEGs were identified following incubation with everolimus and AZA (Table S3).

On each list of DEGs, a pathway analysis was performed with SPIA, which calculated the statistical significance of pathways by considering their topology and the expression levels of their genes. Only for the comparisons between everolimus *vs* control and AZA/everolimus combination *vs* control, significant pathways were detected (Tables 2 and 3). The significant pathways modulated after everolimus treatment (Table 2) were PI3K‐Akt signaling pathway and complement and coagulation cascades. SPIA revealed four significant pathways in the group treated with the synergistic AZA/everolimus combination (Table 3): PI3K‐Akt signaling, neurotrophin signaling pathway, ECM/receptor interaction, and focal adhesion.

**Table 2:** Significant pathways associated with treatment with everolimus by SPIA in MZ‐CRC‐1 cells

Pathway name	pPERT	pNDE	pGFdr
PI3K‐Akt signaling pathway	0.066	0.0013	0.036
Complement and coagulation cascades	0.493	0.0005	0.048

pPERT, probability of observing a total accumulated perturbation of the pathway more extreme than expected by chance; pNDE, probability of obtaining a number of DEGs in the given pathway at least as large as the observed one; pGFdr, false discovery rate for the global *P*‐value from the combination of pPERT and pNDE.

**Table 3:** Significant pathways associated with treatment with everolimus plus AZA by SPIA in MZ‐CRC‐1 cells

Pathway name	pPERT	pNDE	pGFdr
PI3K‐Akt signaling pathway	0.102	0.0002	0.0178
Neurotrophin signaling pathway	0.689	0.0002	0.0308
ECM/receptor interaction	0.115	0.0013	0.0308
Focal adhesion	0.199	0.0018	0.0415

pPERT, probability of observing a total accumulated perturbation of the pathway more extreme than expected by chance; pNDE, probability of obtaining a number of DEGs in the given pathway at least as large as the observed one; pGFdr, false discovery rate for the global *P*‐value from the combination of pPERT and pNDE.

**Figure 6:**
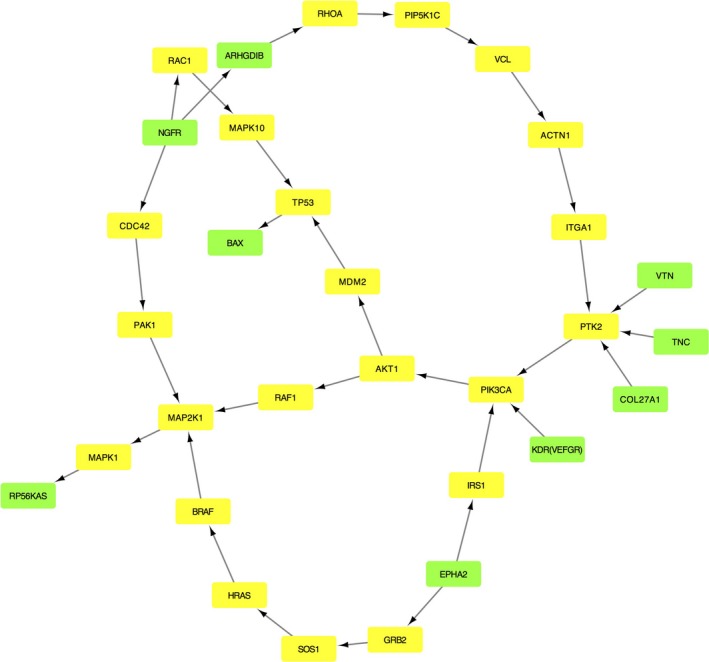
Perturbed pathway module in the group treated with everolimus and 5‐aza‐2′‐deoxycytidine. The green nodes are the DEGs, and the yellow nodes represent the non‐DEGs microarray genes that connect the DEGs. Edges represent interactions between two genes.

The module network obtained by the fusion of the four significant pathways in the group treated with the AZA/everolimus combination is described in Fig. 6. Edges represent interactions between two genes. The list of genes in the module is reported in Table S4. All the DEGs reported in Fig. 6 were overexpressed compared to the untreated control. The SEM multigroup analysis was performed comparing the covariance matrices implied by the model for the treatment data against the control data. The likelihood ratio test had a *P*‐value of 0.002, confirming that the module was differentially regulated between the groups. The perturbed connections identified in this module are reported in Table 4.

**Table 4:** Perturbed connections in the everolimus plus AZA module compared to the untreated control. The estimation of the strength connection and *P*‐value for ‘control’, ‘everolimus plus AZA’, and the ‘difference between everolimus plus AZA *vs* control’ are reported. We have only included the connections where a statistically significant *P* value has been observed for the ‘difference between everolimus plus AZA *vs* control’

Path	Type of process	Control		Everolimus + AZA		Difference	
		Estimate	*P* value	Estimate	*P* value	Estimate	*P* value
MDM2→TP53	Inhibition	0.8271	0.0001	−0.9864	0.0022	−1.8135	0.0000
NGFR→ARHGDIB	Binding/association	0.8935	0.0001	2.7208	0.0001	1.8273	0.0125
EPHA2→IRS1	Activation	−0.5077	0.0079	0.3521	0.2699	0.8598	0.0208
PIP5K1C→VCL	Indirect	−0.6464	0.0237	0.3072	0.0001	0.9536	0.0013
VCL→ACTN1	Binding/association	−0.0985	0.8827	2.4853	0.0002	2.5839	0.0062
ITGA1→PTK2	Binding	−0.3282	0.0206	0.1908	0.2790	0.5190	0.0218
NGFR→RAC1	Activation	−0.9042	0.0180	0.5223	0.0155	1.4265	0.0012
RAC1→MAPK10	Indirect	0.0499	0.6359	0.5799	0.0001	0.5300	0.0033
NGFR→CDC42	Activation	0.2736	0.3068	−1.0567	0.0001	−1.3303	0.0004
PAK1→MAP2K1	Phosphorylation	0.4008	0.0018	−0.0669	0.0998	−0.4678	0.0005

Considering the perturbed edges (Table 4), the links between the NGFR and BAX are found to be significant and intriguing because they are involved in the process of apoptosis (Fig. 6). NGFR was overexpressed during exposure to the AZA/everolimus combination compared to untreated control. In the same condition, the activation of RAC1 (mediated by NGFR) and the indirect interaction RAC1/MAPK10 were found to be stimulated (Table 4). It has been previously reported that the activation of MAPK10 (JNK3), mediated by NGFR/RAC1, can induce apoptosis through the increased expression of TP53 and BAX (Aloyz *et al*., 1998; Kenchappa *et al*., 2010). Although in our model the path TP53→BAX was not significantly perturbed during incubation with the AZA/everolimus combination, an increase in BAX expression was detected during synergistic treatment.

The same procedure was applied for the two pathways (Table 2) previously identified during everolimus treatment. The directed shortest paths between the DEGs in the significant KEGG pathways generated the module reported in Fig. S1. The list of genes in the module is described in Table S5. SEM was used to verify whether globally the strength of connections was statistically different between control and everolimus in the assessed modules, but significant differences were not detected (*P*‐value: 0.1144). Therefore, additional analyses were not performed.

**Figure 7:**
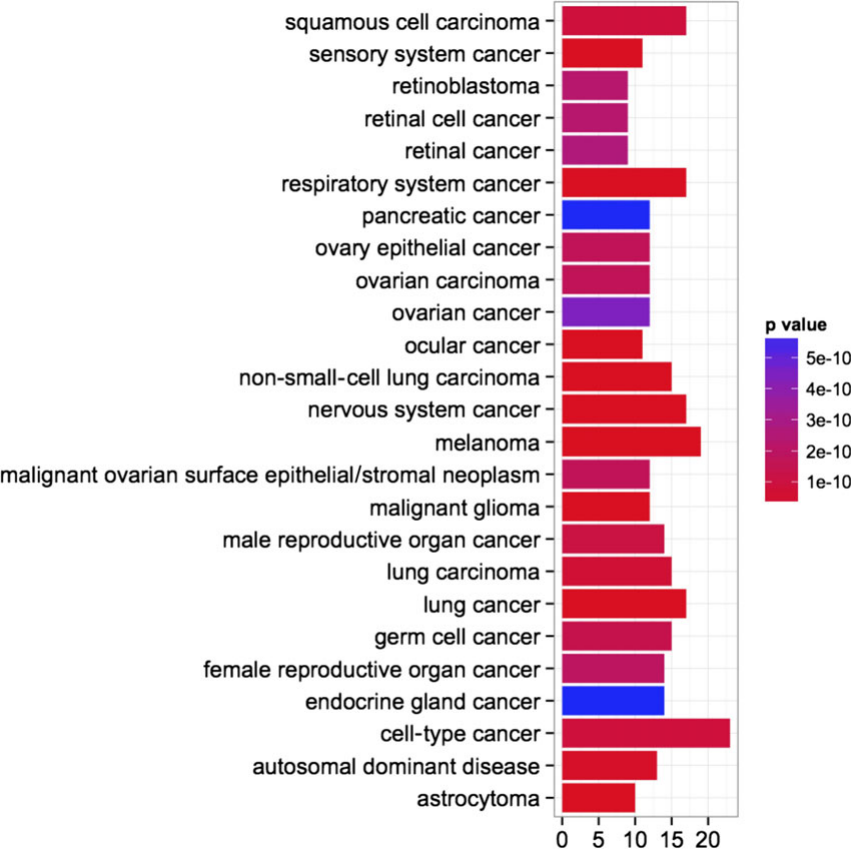
Disease Ontology enrichment analysis for the genes selected in the significant perturbed module, in the group treated with everolimus and 5‐aza‐2′‐deoxycytidine.

An enrichment analysis based on Disease Ontology terms was performed on the genes selected in the module related to the treatment with ‘everolimus plus AZA’ (Fig. 7). Twenty‐four of 25 identified terms were tumors.

### 3.7 Validation of gene expression data

**Figure 8:**
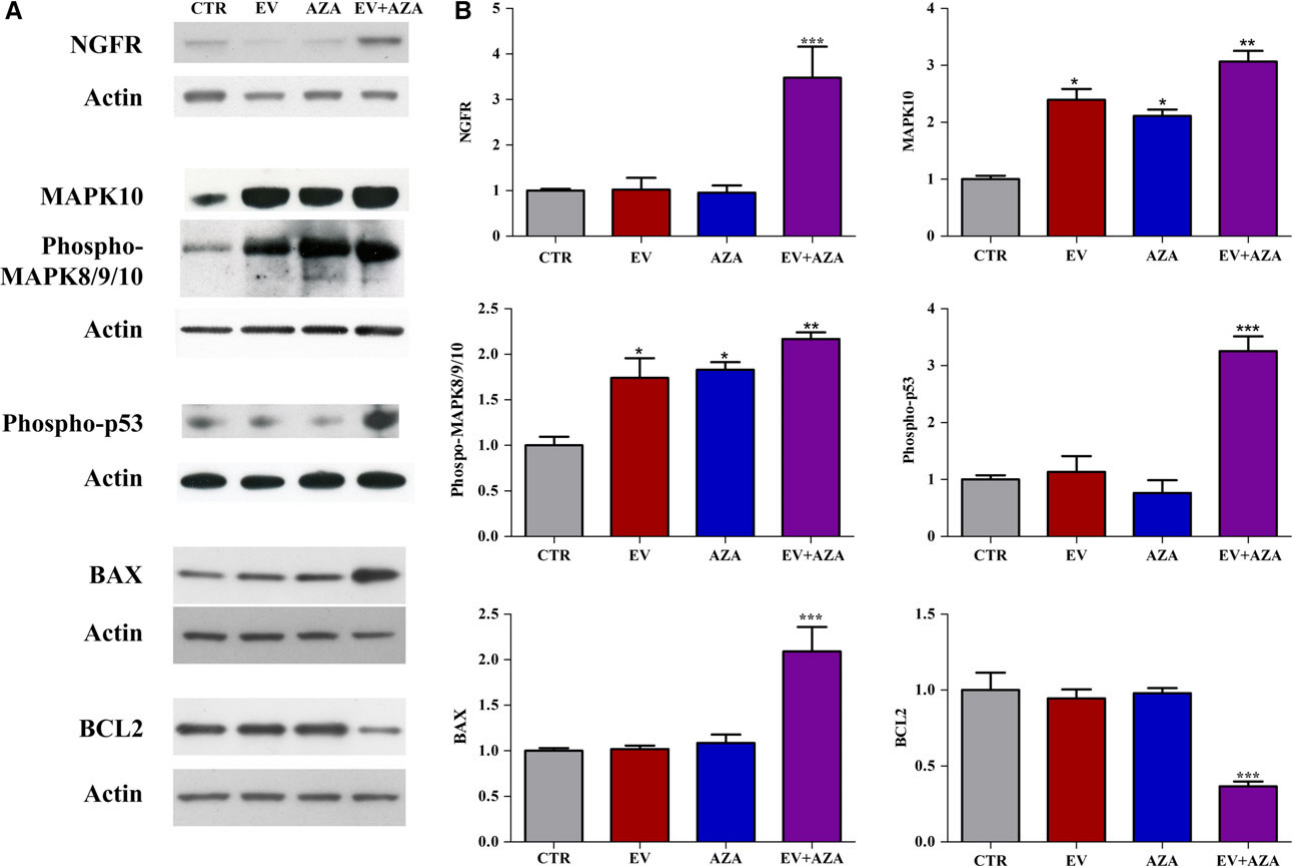
(A) Representative western blot analysis of NGFR, MAPK10, phosphorylated MAPK8/9/10, phosphorylated p53, Bax, and Bcl‐2 performed in MZ‐CRC‐1 cells without (CTR) or after incubation with 2.1 nm everolimus (EV) and/or 1.4 × 10^2^ nm 5‐aza‐2′‐deoxycytidine (AZA). Actin was used as a loading control. (B) Quantification of western blot analysis from at least three independent experiments. *P < 0.05; **P < 0.01; ***P < 0.001.

From the final model of perturbed biological pathways during concomitant incubation with AZA/everolimus combination (Fig. 6), NGFR‐MAPK10‐TP53‐Bax/Bcl‐2 pathway was selected for further analysis on the basis of its strict interaction and modulation of apoptosis, and assessed by western blot (Fig. 8). Indeed, in our system, apoptosis was the cell death mechanism responsible for the synergistic cytotoxic activity of everolimus plus AZA.

A significant increase in NGFR expression has been observed exclusively after 6 days of treatment with the synergistic combination of everolimus plus AZA in MZ‐CRC‐1 cells compared to untreated cells (Fig. 8). Everolimus and AZA alone and in combination increased the protein expression of MAPK10 and phosphorylated MAPK8/9/10; these effects were more pronounced with the combined treatment. In addition, a significant increase in phosphorylated p53 was observed only after incubation with everolimus and AZA combination (Fig. 8). The proapoptotic and antiapoptotic Bax and Bcl‐2, respectively, represent the key proteins involved in mitochondrial pathway of apoptosis. Western blot analysis showed that only the synergistic combination of everolimus plus AZA significantly increased Bax with a concomitant decrease in Bcl2 expression, while there was no significant change in the expression of these proteins after the exposure of MZ‐CRC‐1 cells to everolimus or AZA alone (Fig. 8).

**Figure 9:**
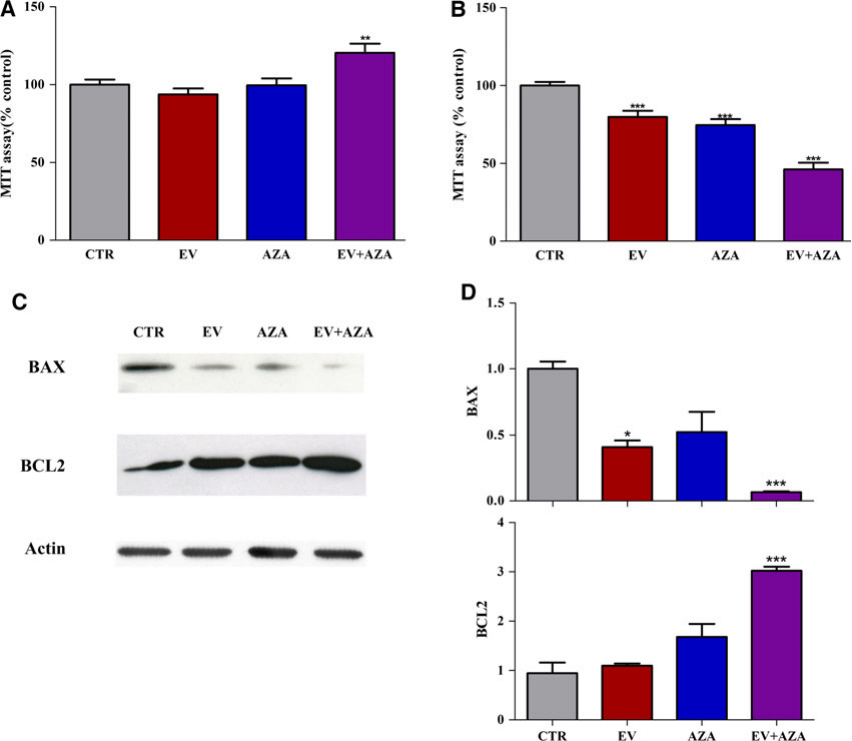
Effect of 2.1 nm everolimus (EV) and/or 1.4 × 10^2^ nm 5‐aza‐2′‐deoxycytidine (AZA) on cell proliferation in MZ‐CRC‐1 cells (A, B) and protein expression of Bax and Bcl‐2 (C, D) after 6 days of treatment, in the presence (A, C, D) or absence (B) of neutralizing antibody against NGFR (1 : 250). (C) Representative western blot analysis. Actin was used as a loading control. (D) Quantification of western blot analysis from at least three independent experiments. *P < 0.05; **P < 0.01; ***P < 0.001.

To verify that the synergistic antitumor activity observed during AZA/everolimus coincubation was indeed due to the increased expression in NGFR, we assessed the effects of both drugs alone or in combination on MZ‐CRC‐1 cell proliferation and protein expression of Bax and Bcl‐2 after 6 days of treatment, in the presence or absence of a neutralizing antibody raised against NGFR (Fig. 9). The concomitant incubation with blocking NGFR antibody counteracted the cytotoxic activity of everolimus and AZA alone, and induced a moderate stimulation in cell proliferation after treatment with AZA/everolimus combination (+20% *vs* control, *P* < 0.01, Fig. 9A). In a parallel set of experiments performed without the incubation with neutralizing antibody against NGFR, the more potent antiproliferative activity after incubation with everolimus plus AZA has been confirmed compared to single drugs (Fig. 9B). During concomitant incubation with neutralizing antibody against NGFR, everolimus plus AZA induced a significant decrease in Bax and increase in Bcl‐2 expression, while these effects were mild with each drug alone (Fig. 9C,D).

### 3.8 Effects of everolimus and AZA on DNA methylation profile

Methylation levels of genes identified in the perturbed everolimus plus AZA module (Fig. 6 and Table S4) were extrapolated from genome‐wide methylation analysis using the Infinium HumanMethylation 450K BeadChip array performed in MZ‐CRC‐1 with or without treatment with everolimus and/or AZA. The array covers 769 probes for these selected 31 genes. Methylation levels for these genes were found to be significantly different among groups (*P* = 3.6 × 10^−5^) (Fig. S2A). A significant reduction in DNA methylation levels has been observed after incubation with AZA alone, similar to that observed after AZA plus everolimus compared to untreated control. The incubation with everolimus alone did not significantly modify DNA methylation levels. These data were confirmed through the density plot showing methylation‐level distributions of untreated and treated samples (Fig. S2B). A similar trend has been observed for NGFR and MAPK10, both methylated genes involved in the NGFR‐MAPK10‐TP53‐Bax/Bcl‐2 pathway (Fig. S2C).

## 4 Discussion

Aberrant promoter methylation of tumor suppressor genes plays a relevant role in the initiation and progression of cancer. In addition, it is being increasingly recognized that aberrant DNA methylation seems to be involved in the development of drug resistance through transcriptional suppression of genes implicated in drug metabolism, apoptosis, cell cycle control, and other biological processes (Hervouet *et al*., 2013). Taking into consideration that DNA methylation is a reversible event unlike genetic mutations, the use of demethylating agents has been recently proposed as a new therapeutic strategy in cancer. Several reports demonstrated the capability of AZA to overcome resistance to chemotherapeutic and biological agents in tumors (Oronsky *et al*., 2014; Vijayaraghavalu *et al*., 2013; Zhang *et al*., 2009). This was encouraging, given that AZA is clinically used for patients with acute myeloid leukemia and myelodysplastic syndromes.

In the current study, combination analysis, based on the Chou–Talalay's method, definitely demonstrated that AZA combined with everolimus was more effective in inhibiting cell proliferation than each agent alone in MTC cells. This cytotoxic activity was highly synergistic through a potent induction of apoptosis in MZ‐CRC‐1 cells, which was the cell line most resistant to everolimus alone. However, in TT cells, combined treatment exhibited both synergistic and antagonistic effects on cell proliferation inhibition, depending on the concentration of the drugs. In MZ‐CRC‐1 cells, while everolimus or AZA alone was barely effective in apoptosis induction, the AZA/everolimus combination doubled the fraction of MTC apoptotic cells compared to untreated controls. Cell cycle analysis showed that everolimus decreased the percentages of MZ‐CRC‐1 cells in G2/M and S phases, but the combination with AZA did not potentiate this effect. Interestingly, the concentrations of AZA and everolimus adopted *in vitro* for the synergistic antiproliferative activity can be readily achieved *in vivo* with acceptable risk benefit (van Groeningen *et al*., 1986; O'Donnell *et al*., 2008). In addition, the *in vitro* toxicity of this combination appeared to be moderate. The cytotoxic activity of everolimus and AZA was lower in HEK‐293 cells, a human embryonic kidney normal cell line, compared to that observed in MZ‐CRC‐1 cells. This is very promising in terms of safety and tolerability for future clinical trials.

Synergistic antiproliferative activity between AZA and everolimus was not related to a direct effect on mTOR activation. In fact, AZA was unable to potentiate the inhibitory activity of everolimus on both mTOR and 4E‐BP1 phosphorylation in MZ‐CRC‐1 cells.

Gene expression analysis revealed potential molecular mechanisms implicated in the synergy of AZA and everolimus in MZ‐CRC‐1 cells. We adopted an innovative bioinformatic pipeline based on SPIA and SEM, recently validated by one of us (Pepe and Grassi, 2014), that allowed us to investigate pathway modules, considering not only deregulated genes but also the connections between the perturbed ones. Several key regulatory genes modulated by the combined treatment of AZA plus everolimus were identified by this approach: PI3K‐Akt signaling, neurotrophin signaling pathway, ECM/receptor interaction, and focal adhesion. Interestingly, all these pathways have a critical role in the regulation of both proliferation and migration/invasion of tumor cells. In addition, a Disease Ontology enrichment analysis, performed on these selected genes, identified 25 biological terms. Twenty‐four of 25 identified terms were tumors, including endocrine gland cancer. The only noncancer item was ‘autosomal dominant disease’. These data further support the pivotal roles of these pathways in the development and progression of MTC. Indeed, MTC is a neuroendocrine tumor and MZ‐CRC‐1 cells harbor the multiple endocrine neoplasia type 2B RET‐M918T mutation, transmitted in this disease as an autosomal dominant trait (Santoro *et al*., 1995).

A perturbed pathway module, associated with the combined treatment of everolimus plus AZA, was generated by the detection and fusion of all shortest paths that put in communication the DEGs (Fig. 6). In this network, the ‘neurotrophin signaling pathway’ appeared to exert a direct influence on the apoptotic machinery through the overexpression of NGFR and the activation of MAPK10‐TP53‐Bax pathway. Neurotrophins are a family of proteins involved in differentiation, plasticity, and survival of neurons and modulate several functions of the neuroendocrine/immune system (Fiore *et al*., 2009). This family includes nerve growth factor (NGF), brain‐derived neurotrophic factor (BDNF), neurotrophins‐3 (NT‐3), neurotrophins‐4/5 (NT‐4/5), and neurotrophins‐6 (NT‐6). Biological activity of these factors is mediated through the activation of Trk tyrosine kinase receptors (TrkA, TrkB, and TrkC) and NGFR (also known as p75 neurotrophin receptor, p75NTR) (Skaper, 2012). In our study, gene expression analysis confirmed the induction of apoptosis through the upregulation of Bax. Bax is a proapoptotic protein antagonized by the antiapoptotic Bcl‐2. The ratio of Bax to Bcl‐2 expression represents a cell death switch, which determines the susceptibility of cells to an apoptotic stimulus. Indeed, a selective decrease in Bax/Bcl‐2 expression represents a common mechanism of drug resistance in tumor cells (Indran *et al*., 2011). We have found a selective increase in the expression of Bax/Bcl‐2 ratio, validated through WB analysis, exclusively after treatment with AZA and everolimus. This effect seems to be associated with the upregulation of NGFR, which has been described to be a tumor suppressor gene in several tumors (Dimaras and Gallie, 2008; Jin *et al*., 2007; Khwaja *et al*., 2004, 2006; Kuchler *et al*., 2011; Kuner and Hertel, 1998; Wang *et al*., 2014; Yang *et al*., 2015; Yuanlong *et al*., 2008), and consecutive activation of MAPK10 and p53.

Although the specific role of NGFR in the tumorigenesis and progression of MTC is unknown, neurotrophin signaling pathway appears to be involved in both preneoplastic thyroid C cell hyperplasia and MTC progression through Trk receptors (McGregor *et al*., 1999). In fact, a cross‐talk between the signal pathways mediated by the Ret proto‐oncogene and Trk receptors has been described (Esposito *et al*., 2008; Peterson and Bogenmann, 2004).

The concomitant incubation with blocking NGFR antibody counteracted the cytotoxic activity of everolimus and AZA alone, and induced a moderate stimulation in cell proliferation and a decrease in Bax/Bcl‐2 ratio expression after treatment with AZA plus everolimus compared to control. This effect could be explained by the block of the proapoptotic pathway mediated by NGFR, while the survival pathways remained activated. These data confirmed that the NGFR overexpression plays a main role in the synergistic cytotoxic activity of these two compounds. Although the mechanism by which the expression of NGFR significantly increased during incubation with everolimus and AZA remains unclear, in colorectal cancer it has been recently reported that NGFR expression was silenced by promoter methylation and that the overexpression of this gene significantly inhibited cell proliferation, invasion and stimulated cell apoptosis (Yang *et al*., 2015). In our model, the overexpression of NGFR appears not to be related to a direct epigenetic effect on NGFR gene. In fact, during incubation of MZ‐CRC‐1 cells with AZA, we found a significant decrease in DNA methylation levels of NGFR, comparable to that observed after everolimus plus AZA, while NGFR expression increased exclusively after combined treatment.

Intriguingly, the synergistic cytotoxic activity of everolimus/AZA combination was more relevant in MZ‐CRC‐1 (harboring RET‐M918T mutation) than in TT (harboring RET‐C634W mutation) cells (Table 1). We cannot exclude that the type of RET genetic alteration may have a role in the different antitumor activity observed in both cell lines. In fact, Gild *et al*. (2013) demonstrated that oncogenic RET regulates mTOR activity in MZ‐CRC‐1 and TT cell lines. Combined incubation with RET and mTOR inhibitors (AST487 and INK128, respectively) at low concentrations cooperated to inhibit mTOR signaling and cell growth, through the induction of apoptosis, in both MTC cell lines. In addition, a recent study found that methylation profiles relate closely to RET mutational status in MTC, and the most distinctive methylome was observed for RET‐M918T‐positive tumors (Mancikova *et al*., 2017).

In conclusion, we described for the first time a synergistic cytotoxic activity combining AZA with everolimus in MTC. This effect occurred through the overactivation of NGFR‐MAPK10‐TP53‐Bax/Bcl‐2 pathway and the induction of apoptosis. These data provide a new therapeutic scenario in MTC and probably in other neuroendocrine tumors, where therapy with everolimus is currently approved.

## Author contributions

GV, AMDB, LJH, MC and LP conceived and designed the project; AD, DG, ESG, MOB, GG, MCC, GG and GM acquired the data; GV, AD, DP and DG analyzed and interpreted the data; GV, AD, DP and DG wrote the manuscript.

## Supporting information


**Fig. S1.** Perturbed pathway module in the group treated with everolimus.
**Fig. S2.** (A) Box‐and‐whisker‐plots showing global methylation levels measured in 769 probes within 31 genes, selected from the perturbed module reported in Figure 6, in MZ‐CRC‐1 cells without (CTR) or with everolimus (EV) and/or 5‐aza‐2′deoxycytidine (AZA). (B) Density plot showing methylation level distributions of treated and untreated samples. (C) Box‐and‐whisker‐plots showing global methylation levels of NGFR, MAPK10, TP53, BAX and BCL2 genes in treated and untreated samples.
**Table S1.** Differentially expressed genes (DEGs) following incubation with everolimus *vs* untreated control identified by Significance Analysis of Microarray (SAM), using a delta value of 0.46. Fold change (FC).
**Table S2.** Differentially expressed genes (DEGs) following incubation with AZA *vs* untreated control identified by Significance Analysis of Microarray (SAM), using a delta value of 0.102. Fold change (FC).
**Table S3.** Differentially expressed genes (DEGs) following incubation with everolimus plus AZA *vs* untreated control identified by Significance Analysis of Microarray (SAM), using a delta value of 0.46. Fold change (FC).
**Table S4.** Genes in the perturbed everolimus plus AZA module. The gene ID, the gene symbol and if the gene is DEG (1) or not DEG (0) are reported.
**Table S5.** Genes in the perturbed everolimus module.
