# Supplementary material for: Synergistic activity of everolimus and 5‐aza‐2′‐deoxycytidine in medullary thyroid carcinoma cell lines
Source: Mol Oncol. 2017 Jun 21;11(8):1007–22. doi: 10.1002/1878-0261.12070 (PMC5537710; doi:10.1002/1878-0261.12070)

**Supplementary Table 1.** Differentially expressed genes (DEGs) following incubation with everolimus *vs* untreated control identified by Significance Analysis of Microarray (SAM), using a delta value of 0.46. Fold change (FC)

| **Gene ID** | **Gene symbol** | **Gene Description** | **Log_2_ FC** |
| --- | --- | --- | --- |
| 84448 | ABLIM2 | actin binding LIM protein family, member 2 | 0.41 |
| 401036 | ASB18 | ankyrin repeat and SOCS box-containing 18 | 0.54 |
| 80017 | C14orf159 | chromosome 14 open reading frame 159 | 0.43 |
| 574036 | C1orf133 | chromosome 1 open reading frame 133 | -0.66 |
| 92291 | CAPN13 | calpain 13 | 0.71 |
| 147381 | CBLN2 | cerebellin 2 precursor | -0.79 |
| 1082 | CGB | chorionic gonadotropin, beta polypeptide | 0.54 |
| 93659 | CGB5 | chorionic gonadotropin, beta polypeptide 5 | 0.71 |
| 94115 | CGB8 | chorionic gonadotropin, beta polypeptide 8 | 0.65 |
| 400916 | CHCHD10 | coiled-coil-helix-coiled-coil-helix domain containing 10 | 0.60 |
| 10815 | CPLX1 | complexin 1 | 0.52 |
| 1381 | CRABP1 | cellular retinoic acid binding protein 1 | 0.49 |
| 54541 | DDIT4 | DNA-damage-inducible transcript 4 | -0.49 |
| 1666 | DECR1 | 2,4-dienoyl CoA reductase 1, mitochondrial | -0.51 |
| 1749 | DLX5 | distal-less homeobox 5 | -0.88 |
| 93099 | DMKN | dermokine | 0.62 |
| 55556 | ENOSF1 | enolase superfamily member 1 | 0.40 |
| 1969 | EPHA2 | EPH receptor A2 | 0.88 |
| 2153 | F5 | coagulation factor V (proaccelerin, labile factor) | -0.84 |
| 84141 | FAM176A | family with sequence similarity 176, member A | -0.56 |
| 220382 | FAM181B | family with sequence similarity 181, member B | -0.61 |
| 54757 | FAM20A | family with sequence similarity 20, member A | 0.63 |
| 84961 | FBXL20 | F-box and leucine-rich repeat protein 20 | 0.51 |
| 401388 | FLJ42280 | hypothetical LOC401388 | -0.53 |
| 252995 | FNDC5 | fibronectin type III domain containing 5 | 0.57 |
| 221395 | GPR116 | G protein-coupled receptor 116 | 0.49 |
| 2827 | GPR3 | G protein-coupled receptor 3 | 0.49 |
| 2905 | GRIN2C | glutamate receptor, ionotropic, N-methyl D-aspartate 2C | 0.47 |
| 253012 | HEPACAM2 | HEPACAM family member 2 | -0.56 |
| 3006 | HIST1H1C | histone cluster 1, H1c | -0.85 |
| 8349 | HIST2H2BE | histone cluster 2, H2be | 0.39 |
| 3375 | IAPP | islet amyloid polypeptide | 2.31 |
| 3485 | IGFBP2 | insulin-like growth factor binding protein 2, 36kDa | 1.07 |
| 3600 | IL15 | interleukin 15 | -0.48 |
| 23421 | ITGB3BP | integrin beta 3 binding protein (beta3-endonexin) | -0.55 |
| 55600 | ITLN1 | intelectin 1 (galactofuranose binding) | 0.82 |
| 142683 | ITLN2 | intelectin 2 | 1.21 |
| 3791 | KDR | kinase insert domain receptor (a type III receptor tyrosine kinase) | 0.55 |
| 3872 | KRT17 | keratin 17; keratin 17 pseudogene 3 | 0.54 |
| 84894 | LINGO1 | leucine rich repeat and Ig domain containing 1 | 0.50 |
| 645967 | LOC645967 | hypothetical LOC645967 | 0.70 |
| 23764 | MAFF | v-maf musculoaponeurotic fibrosarcoma oncogene homolog F (avian) | 0.58 |
| 29116 | MYLIP | myosin regulatory light chain interacting protein | 0.41 |
| 4745 | NELL1 | NEL-like 1 (chicken) | 0.70 |
| 9891 | NUAK1 | NUAK family, SNF1-like kinase, 1 | 0.37 |
| 58495 | OVOL2 | ovo-like 2 (Drosophila) | -0.69 |
| 113791 | PIK3IP1 | phosphoinositide-3-kinase interacting protein 1 | -0.49 |
| 5292 | PIM1 | pim-1 oncogene | 0.53 |
| 10687 | PNMA2 | paraneoplastic antigen MA2 | 0.47 |
| 10891 | PPARGC1A | peroxisome proliferator-activated receptor gamma, coactivator 1 alpha | 0.67 |
| 151242 | PPP1R1C | protein phosphatase 1, regulatory (inhibitor) subunit 1C | -0.68 |
| 7837 | PXDN | peroxidasin homolog (Drosophila) | 0.60 |
| 6201 | RPS7 | ribosomal protein S7 | 0.43 |
| 6319 | SCD | stearoyl-CoA desaturase (delta-9-desaturase) | -0.81 |
| 6338 | SCNN1B | sodium channel, nonvoltage-gated 1, beta | 0.87 |
| 3053 | SERPIND1 | serpin peptidase inhibitor, clade D (heparin cofactor), member 1 | 0.41 |
| 143686 | SESN3 | sestrin 3 | -0.43 |
| 387914 | SHISA2 | shisa homolog 2 (Xenopus laevis) | 0.79 |
| 57619 | SHROOM3 | shroom family member 3 | 0.56 |
| 201780 | SLC10A4 | solute carrier family 10 (sodium/bile acid cotransporter family), member 4 | -0.59 |
| 3431 | SP110 | SP110 nuclear body protein | -0.56 |
| 6883 | TAF12 | TAF12 RNA polymerase II, TATA box binding protein (TBP)-associated factor, 20kDa | 0.44 |
| 8148 | TAF15 | TAF15 RNA polymerase II, TATA box binding protein (TBP)-associated factor, 68kDa | 0.51 |
| 54997 | TESC | tescalcin | 0.72 |
| 7056 | THBD | thrombomodulin | 0.58 |
| 392636 | TMEM195 | transmembrane protein 195 | -0.43 |
| 26022 | TMEM98 | similar to transmembrane protein 98; transmembrane protein 98 | -0.57 |
| 3371 | TNC | tenascin C | 0.61 |
| 142940 | TRUB1 | TruB pseudouridine (psi) synthase homolog 1 (E. coli) | -0.42 |
| 8237 | USP11 | ubiquitin specific peptidase 11 | 0.40 |
| 7448 | VTN | vitronectin | 0.61 |
| 54361 | WNT4 | wingless-type MMTV integration site family, member 4 | 0.60 |

**Supplementary Table 2.** Differentially expressed genes (DEGs) following incubation with AZA *vs* untreated control identified by Significance Analysis of Microarray (SAM), using a delta value of 0.102. Fold change (FC)

| **Gene ID** | **Gene symbol** | **Gene Description** | **Log_2_ FC** |
| --- | --- | --- | --- |
| 8277 | TKTL1 | transketolase-like 1 | 2.40 |
| 9518 | GDF15 | growth differentiation factor 15 | 1.10 |
| 397 | ARHGDIB | Rho GDP dissociation inhibitor (GDI) beta | 0.96 |
| 3371 | TNC | tenascin C | 0.83 |
| 1056 | CEL | carboxyl ester lipase | 0.72 |
| 221395 | GPR116 | G protein-coupled receptor 116 | 0.74 |
| 1969 | EPHA2 | EPH receptor A2 | 0.76 |
| 59 | ACTA2 | actin, alpha 2, smooth muscle, aorta | 0.65 |
| 3880 | KRT19 | keratin 19 | 0.63 |
| 6275 | S100A4 | S100 calcium binding protein A4 | 0.69 |
| 7114 | TMSB4X | thymosin beta 4, X-linked | 0.60 |
| 283120 | H19 | H19, imprinted maternally expressed transcript | 0.64 |
| 7056 | THBD | thrombomodulin | 0.62 |
| 3956 | LGALS1 | lectin, galactoside-binding, soluble, 1 | 0.67 |
| 8797 | TNFRSF10A | tumor necrosis factor receptor superfamily, member 10a | 0.60 |
| 7117 | TMSL3 | thymosin-like 3 | 0.61 |

**Supplementary Table 3.** Differentially expressed genes (DEGs) following incubation with everolimus plus AZA *vs* untreated control identified by Significance Analysis of Microarray (SAM), using a delta value of 0.46. Fold change (FC)

| **Gene ID** | **Gene symbol** | **Gene Description** | **Log_2_ FC** |
| --- | --- | --- | --- |
| 59 | [acta2](http://www.ncbi.nlm.nih.gov/entrez/query.fcgi?db=gene&cmd=Retrieve&dopt=Graphics&list_uids=acta2) | actin, alpha 2, smooth muscle, aorta | 0.75 |
| 397 | [arhgdib](http://www.ncbi.nlm.nih.gov/entrez/query.fcgi?db=gene&cmd=Retrieve&dopt=Graphics&list_uids=arhgdib) | Rho GDP dissociation inhibitor (GDI) beta | 0.89 |
| 581 | [BAX](http://www.ncbi.nlm.nih.gov/entrez/query.fcgi?db=gene&cmd=Retrieve&dopt=Graphics&list_uids=BAX) | BCL2-associated X protein | 0.59 |
| 820 | [CAMP](http://www.ncbi.nlm.nih.gov/entrez/query.fcgi?db=gene&cmd=Retrieve&dopt=Graphics&list_uids=CAMP) | cathelicidin antimicrobial peptide | 0.63 |
| 929 | [CD14](http://www.ncbi.nlm.nih.gov/entrez/query.fcgi?db=gene&cmd=Retrieve&dopt=Graphics&list_uids=CD14) | CD14 molecule | 0.52 |
| 1056 | [Cel](http://www.ncbi.nlm.nih.gov/entrez/query.fcgi?db=gene&cmd=Retrieve&dopt=Graphics&list_uids=Cel) | carboxyl ester lipase (bile salt-stimulated lipase) | 0.76 |
| 1082 | [CGB](http://www.ncbi.nlm.nih.gov/entrez/query.fcgi?db=gene&cmd=Retrieve&dopt=Graphics&list_uids=CGB) | chorionic gonadotropin, beta polypeptide | 0.56 |
| 1382 | [crabp2](http://www.ncbi.nlm.nih.gov/entrez/query.fcgi?db=gene&cmd=Retrieve&dopt=Graphics&list_uids=crabp2) | cellular retinoic acid binding protein 2 | 0.43 |
| 1749 | [DLX5](http://www.ncbi.nlm.nih.gov/entrez/query.fcgi?db=gene&cmd=Retrieve&dopt=Graphics&list_uids=DLX5) | distal-less homeobox 5 | -0.91 |
| 1893 | [Ecm1](http://www.ncbi.nlm.nih.gov/entrez/query.fcgi?db=gene&cmd=Retrieve&dopt=Graphics&list_uids=Ecm1) | extracellular matrix protein 1 | 0.53 |
| 1969 | [EPHA2](http://www.ncbi.nlm.nih.gov/entrez/query.fcgi?db=gene&cmd=Retrieve&dopt=Graphics&list_uids=EPHA2) | EPH receptor A2 | 0.84 |
| 2905 | [grin2c](http://www.ncbi.nlm.nih.gov/entrez/query.fcgi?db=gene&cmd=Retrieve&dopt=Graphics&list_uids=grin2c) | glutamate receptor, ionotropic, N-methyl D-aspartate 2C | 0.52 |
| 3053 | [serpind1](http://www.ncbi.nlm.nih.gov/entrez/query.fcgi?db=gene&cmd=Retrieve&dopt=Graphics&list_uids=serpind1) | serpin peptidase inhibitor, clade D (heparin cofactor), member 1 | 0.67 |
| 3371 | [TNC](http://www.ncbi.nlm.nih.gov/entrez/query.fcgi?db=gene&cmd=Retrieve&dopt=Graphics&list_uids=TNC) | tenascin C | 0.92 |
| 3375 | [Iapp](http://www.ncbi.nlm.nih.gov/entrez/query.fcgi?db=gene&cmd=Retrieve&dopt=Graphics&list_uids=Iapp) | islet amyloid polypeptide | 1.90 |
| 3485 | [igfbp2](http://www.ncbi.nlm.nih.gov/entrez/query.fcgi?db=gene&cmd=Retrieve&dopt=Graphics&list_uids=igfbp2) | insulin-like growth factor binding protein 2, 36kDa | 0.98 |
| 3576 | [IL8](http://www.ncbi.nlm.nih.gov/entrez/query.fcgi?db=gene&cmd=Retrieve&dopt=Graphics&list_uids=IL8) | interleukin 8 | 0.75 |
| 3791 | [KDR](http://www.ncbi.nlm.nih.gov/entrez/query.fcgi?db=gene&cmd=Retrieve&dopt=Graphics&list_uids=KDR) | kinase insert domain receptor (a type III receptor tyrosine kinase) | 0.62 |
| 3872 | [KRT17P3](http://www.ncbi.nlm.nih.gov/entrez/query.fcgi?db=gene&cmd=Retrieve&dopt=Graphics&list_uids=KRT17P3) | keratin 17; keratin 17 pseudogene 3 | 0.73 |
| 4703 | [NEB](http://www.ncbi.nlm.nih.gov/entrez/query.fcgi?db=gene&cmd=Retrieve&dopt=Graphics&list_uids=NEB) | Nebulin | -0.60 |
| 4745 | [Nell1](http://www.ncbi.nlm.nih.gov/entrez/query.fcgi?db=gene&cmd=Retrieve&dopt=Graphics&list_uids=Nell1) | NEL-like 1 (chicken) | 0.56 |
| 4804 | [Ngfr](http://www.ncbi.nlm.nih.gov/entrez/query.fcgi?db=gene&cmd=Retrieve&dopt=Graphics&list_uids=Ngfr) | nerve growth factor receptor (TNFR superfamily, member 16) | 0.59 |
| 4851 | [notch1](http://www.ncbi.nlm.nih.gov/entrez/query.fcgi?db=gene&cmd=Retrieve&dopt=Graphics&list_uids=notch1) | Notch homolog 1, translocation-associated (Drosophila) | 0.67 |
| 5630 | [prpH](http://www.ncbi.nlm.nih.gov/entrez/query.fcgi?db=gene&cmd=Retrieve&dopt=Graphics&list_uids=prpH) | Peripherin | 0.40 |
| 6338 | [SCNN1B](http://www.ncbi.nlm.nih.gov/entrez/query.fcgi?db=gene&cmd=Retrieve&dopt=Graphics&list_uids=SCNN1B) | sodium channel, nonvoltage-gated 1, beta | 0.72 |
| 6623 | [SNCG](http://www.ncbi.nlm.nih.gov/entrez/query.fcgi?db=gene&cmd=Retrieve&dopt=Graphics&list_uids=SNCG) | synuclein, gamma (breast cancer-specific protein 1) | 0.36 |
| 6920 | [TCEA3](http://www.ncbi.nlm.nih.gov/entrez/query.fcgi?db=gene&cmd=Retrieve&dopt=Graphics&list_uids=TCEA3) | transcription elongation factor A (SII), 3 | 0.43 |
| 7056 | [THBD](http://www.ncbi.nlm.nih.gov/entrez/query.fcgi?db=gene&cmd=Retrieve&dopt=Graphics&list_uids=THBD) | Thrombomodulin | 0.76 |
| 7114 | [TMSL1](http://www.ncbi.nlm.nih.gov/entrez/query.fcgi?db=gene&cmd=Retrieve&dopt=Graphics&list_uids=TMSL1) | thymosin-like 1 (pseudogene) | 0.52 |
| 7117 | [TMSL3](http://www.ncbi.nlm.nih.gov/entrez/query.fcgi?db=gene&cmd=Retrieve&dopt=Graphics&list_uids=TMSL3) | thymosin-like 3 | 0.64 |
| 7448 | [vtn](http://www.ncbi.nlm.nih.gov/entrez/query.fcgi?db=gene&cmd=Retrieve&dopt=Graphics&list_uids=vtn) | Vitronectin | 0.70 |
| 7783 | [ZP2](http://www.ncbi.nlm.nih.gov/entrez/query.fcgi?db=gene&cmd=Retrieve&dopt=Graphics&list_uids=ZP2) | zona pellucida glycoprotein 2 (sperm receptor) | 0.59 |
| 7837 | [PXDN](http://www.ncbi.nlm.nih.gov/entrez/query.fcgi?db=gene&cmd=Retrieve&dopt=Graphics&list_uids=PXDN) | peroxidasin homolog (Drosophila) | 0.65 |
| 8277 | [TKTL1](http://www.ncbi.nlm.nih.gov/entrez/query.fcgi?db=gene&cmd=Retrieve&dopt=Graphics&list_uids=TKTL1) | transketolase-like 1 | 1.88 |
| 8406 | [SRPX](http://www.ncbi.nlm.nih.gov/entrez/query.fcgi?db=gene&cmd=Retrieve&dopt=Graphics&list_uids=SRPX) | sushi-repeat-containing protein, X-linked | 0.60 |
| 8459 | [TPST2](http://www.ncbi.nlm.nih.gov/entrez/query.fcgi?db=gene&cmd=Retrieve&dopt=Graphics&list_uids=TPST2) | tyrosylprotein sulfotransferase 2 | 0.37 |
| 9024 | [BRSK2](http://www.ncbi.nlm.nih.gov/entrez/query.fcgi?db=gene&cmd=Retrieve&dopt=Graphics&list_uids=BRSK2) | BR serine/threonine kinase 2 | 0.51 |
| 9252 | [RPS6KA5](http://www.ncbi.nlm.nih.gov/entrez/query.fcgi?db=gene&cmd=Retrieve&dopt=Graphics&list_uids=RPS6KA5) | ribosomal protein S6 kinase, 90kDa, polypeptide 5 | 0.62 |
| 9271 | [PIWIL1](http://www.ncbi.nlm.nih.gov/entrez/query.fcgi?db=gene&cmd=Retrieve&dopt=Graphics&list_uids=PIWIL1) | piwi-like 1 (Drosophila) | 0.42 |
| 9518 | [Gdf15](http://www.ncbi.nlm.nih.gov/entrez/query.fcgi?db=gene&cmd=Retrieve&dopt=Graphics&list_uids=Gdf15) | growth differentiation factor 15 | 1.14 |
| 10866 | [hcp5](http://www.ncbi.nlm.nih.gov/entrez/query.fcgi?db=gene&cmd=Retrieve&dopt=Graphics&list_uids=hcp5) | HLA complex P5 | 0.43 |
| 22846 | [Vash1](http://www.ncbi.nlm.nih.gov/entrez/query.fcgi?db=gene&cmd=Retrieve&dopt=Graphics&list_uids=Vash1) | vasohibin 1 | 0.37 |
| 23017 | [FAIM2](http://www.ncbi.nlm.nih.gov/entrez/query.fcgi?db=gene&cmd=Retrieve&dopt=Graphics&list_uids=FAIM2) | Fas apoptotic inhibitory molecule 2 | 0.50 |
| 23764 | [Maff](http://www.ncbi.nlm.nih.gov/entrez/query.fcgi?db=gene&cmd=Retrieve&dopt=Graphics&list_uids=Maff) | v-maf musculoaponeurotic fibrosarcoma oncogene homolog F (avian) | 0.42 |
| 27163 | [NAAA](http://www.ncbi.nlm.nih.gov/entrez/query.fcgi?db=gene&cmd=Retrieve&dopt=Graphics&list_uids=NAAA) | N-acylethanolamine acid amidase | -0.60 |
| 29116 | [MYLIP](http://www.ncbi.nlm.nih.gov/entrez/query.fcgi?db=gene&cmd=Retrieve&dopt=Graphics&list_uids=MYLIP) | myosin regulatory light chain interacting protein | 0.44 |
| 51440 | [HPCAL4](http://www.ncbi.nlm.nih.gov/entrez/query.fcgi?db=gene&cmd=Retrieve&dopt=Graphics&list_uids=HPCAL4) | hippocalcin like 4 | 0.52 |
| 54361 | [WNT4](http://www.ncbi.nlm.nih.gov/entrez/query.fcgi?db=gene&cmd=Retrieve&dopt=Graphics&list_uids=WNT4) | wingless-type MMTV integration site family, member 4 | 0.79 |
| 54757 | [fam20a](http://www.ncbi.nlm.nih.gov/entrez/query.fcgi?db=gene&cmd=Retrieve&dopt=Graphics&list_uids=fam20a) | family with sequence similarity 20, member A | 0.57 |
| 54830 | [NUP62CL](http://www.ncbi.nlm.nih.gov/entrez/query.fcgi?db=gene&cmd=Retrieve&dopt=Graphics&list_uids=NUP62CL) | nucleoporin 62kDa C-terminal like | 0.39 |
| 54997 | [TESC](http://www.ncbi.nlm.nih.gov/entrez/query.fcgi?db=gene&cmd=Retrieve&dopt=Graphics&list_uids=TESC) | Tescalcin | 0.76 |
| 55049 | [C19orf60](http://www.ncbi.nlm.nih.gov/entrez/query.fcgi?db=gene&cmd=Retrieve&dopt=Graphics&list_uids=C19orf60) | chromosome 19 open reading frame 60 | 0.51 |
| 55600 | [Itln1](http://www.ncbi.nlm.nih.gov/entrez/query.fcgi?db=gene&cmd=Retrieve&dopt=Graphics&list_uids=Itln1) | intelectin 1 (galactofuranose binding) | 0.72 |
| 57146 | [TMEM159](http://www.ncbi.nlm.nih.gov/entrez/query.fcgi?db=gene&cmd=Retrieve&dopt=Graphics&list_uids=TMEM159) | transmembrane protein 159 | 0.44 |
| 57619 | [SHROOM3](http://www.ncbi.nlm.nih.gov/entrez/query.fcgi?db=gene&cmd=Retrieve&dopt=Graphics&list_uids=SHROOM3) | shroom family member 3 | 0.54 |
| 58495 | [OVOL2](http://www.ncbi.nlm.nih.gov/entrez/query.fcgi?db=gene&cmd=Retrieve&dopt=Graphics&list_uids=OVOL2) | ovo-like 2 (Drosophila) | -0.81 |
| 79190 | [IRX6](http://www.ncbi.nlm.nih.gov/entrez/query.fcgi?db=gene&cmd=Retrieve&dopt=Graphics&list_uids=IRX6) | iroquois homeobox 6 | 0.61 |
| 83741 | [TFAP2D](http://www.ncbi.nlm.nih.gov/entrez/query.fcgi?db=gene&cmd=Retrieve&dopt=Graphics&list_uids=TFAP2D) | transcription factor AP-2 delta (activating enhancer binding protein 2 delta) | 0.34 |
| 85301 | [Col27a1](http://www.ncbi.nlm.nih.gov/entrez/query.fcgi?db=gene&cmd=Retrieve&dopt=Graphics&list_uids=Col27a1) | collagen, type XXVII, alpha 1 | 0.51 |
| 92291 | [CAPN13](http://www.ncbi.nlm.nih.gov/entrez/query.fcgi?db=gene&cmd=Retrieve&dopt=Graphics&list_uids=CAPN13) | calpain 13 | 0.72 |
| 93659 | [CGB](http://www.ncbi.nlm.nih.gov/entrez/query.fcgi?db=gene&cmd=Retrieve&dopt=Graphics&list_uids=CGB) | chorionic gonadotropin, beta polypeptide 5; chorionic gonadotropin, beta polypeptide; chorionic gonadotropin, beta polypeptide 8 | 0.66 |
| 94115 | [CGB](http://www.ncbi.nlm.nih.gov/entrez/query.fcgi?db=gene&cmd=Retrieve&dopt=Graphics&list_uids=CGB) | chorionic gonadotropin, beta polypeptide 5; chorionic gonadotropin, beta polypeptide; chorionic gonadotropin, beta polypeptide 8 | 0.86 |
| 116151 | [c20orf108](http://www.ncbi.nlm.nih.gov/entrez/query.fcgi?db=gene&cmd=Retrieve&dopt=Graphics&list_uids=c20orf108) | chromosome 20 open reading frame 108 | 0.43 |
| 142683 | [ITLN2](http://www.ncbi.nlm.nih.gov/entrez/query.fcgi?db=gene&cmd=Retrieve&dopt=Graphics&list_uids=ITLN2) | intelectin 2 | 1.34 |
| 147381 | [CBLN2](http://www.ncbi.nlm.nih.gov/entrez/query.fcgi?db=gene&cmd=Retrieve&dopt=Graphics&list_uids=CBLN2) | cerebellin 2 precursor | -0.74 |
| 221395 | [Gpr116](http://www.ncbi.nlm.nih.gov/entrez/query.fcgi?db=gene&cmd=Retrieve&dopt=Graphics&list_uids=Gpr116) | G protein-coupled receptor 116 | 0.90 |
| 252995 | [FNDC5](http://www.ncbi.nlm.nih.gov/entrez/query.fcgi?db=gene&cmd=Retrieve&dopt=Graphics&list_uids=FNDC5) | fibronectin type III domain containing 5 | 0.63 |
| 341405 | [ANKRD33](http://www.ncbi.nlm.nih.gov/entrez/query.fcgi?db=gene&cmd=Retrieve&dopt=Graphics&list_uids=ANKRD33) | ankyrin repeat domain 33 | 0.46 |
| 387914 | [SHISA2](http://www.ncbi.nlm.nih.gov/entrez/query.fcgi?db=gene&cmd=Retrieve&dopt=Graphics&list_uids=SHISA2) | shisa homolog 2 (Xenopus laevis) | 0.64 |
| 401036 | [ASB18](http://www.ncbi.nlm.nih.gov/entrez/query.fcgi?db=gene&cmd=Retrieve&dopt=Graphics&list_uids=ASB18) | ankyrin repeat and SOCS box-containing 18 | 0.58 |
| 404093 | [CUEDC1](http://www.ncbi.nlm.nih.gov/entrez/query.fcgi?db=gene&cmd=Retrieve&dopt=Graphics&list_uids=CUEDC1) | CUE domain containing 1 | 0.55 |
| 652683 or 728695 | SPANXB1 | SPANX family, member B1 | 0.53 |
| 100130449 | [PP14571](http://www.ncbi.nlm.nih.gov/entrez/query.fcgi?db=gene&cmd=Retrieve&dopt=Graphics&list_uids=PP14571) | similar to hCG1777210 | 0.34 |
| 100134134 | LOC100134134 | LOC100134134 similar to peroxidasin homolog | 0.65 |

**Supplementary Table 4.** Genes in the perturbed everolimus plus AZA module. The gene ID, the gene symbol and if the gene is DEG (1) or not DEG (0) are reported.

| **Gene ID** | **Gene symbol** | **DEG 1/0** |
| --- | --- | --- |
| 3371 | TNC | 1 |
| 5747 | PTK2 | 0 |
| 5290 | PIK3CA | 0 |
| 207 | AKT1 | 0 |
| 4193 | MDM2 | 0 |
| 7157 | TP53 | 0 |
| 4804 | NGFR | 1 |
| 5894 | RAF1 | 0 |
| 5604 | MAP2K1 | 0 |
| 5594 | MAPK1 | 0 |
| 1969 | EPHA2 | 1 |
| 3667 | IRS1 | 0 |
| 2885 | GRB2 | 0 |
| 6654 | SOS1 | 0 |
| 3265 | HRAS | 0 |
| 673 | BRAF | 0 |
| 7448 | VTN | 1 |
| 397 | ARHGDIB | 1 |
| 387 | RHOA | 0 |
| 23396 | PIP5K1C | 0 |
| 7414 | VCL | 0 |
| 87 | ACTN1 | 0 |
| 3672 | ITGA1 | 0 |
| 5879 | RAC1 | 0 |
| 5602 | MAPK10 | 0 |
| 998 | CDC42 | 0 |
| 5058 | PAK1 | 0 |
| 85301 | COL27A1 | 0 |
| 3791 | KDR | 1 |
| 581 | BAX | 1 |
| 9252 | RPS6KA5 | 1 |

**Supplementary Table 5.** Genes in the perturbed everolimus module. The gene ID, the gene symbol and if the gene is DEG (1) or not DEG (0) are reported.

| **Gene ID** | **Gene symbol** | **DEG 1/0** |
| --- | --- | --- |
| 7056 | THBD | 1 |
| 5624 | PROC | 0 |
| 3053 | SERPIND1 | 1 |
| 2147 | F2 | 0 |
| 2158 | F9 | 0 |
| 2159 | F10 | 0 |
| 2153 | F5 | 1 |

**Supplementary Figure 1.** Perturbed pathway module in the group treated with everolimus. The green nodes are the DEGs, the yellow nodes represent the not DEGs microarray genes that connect the DEGs. Edges represent interactions between two genes.


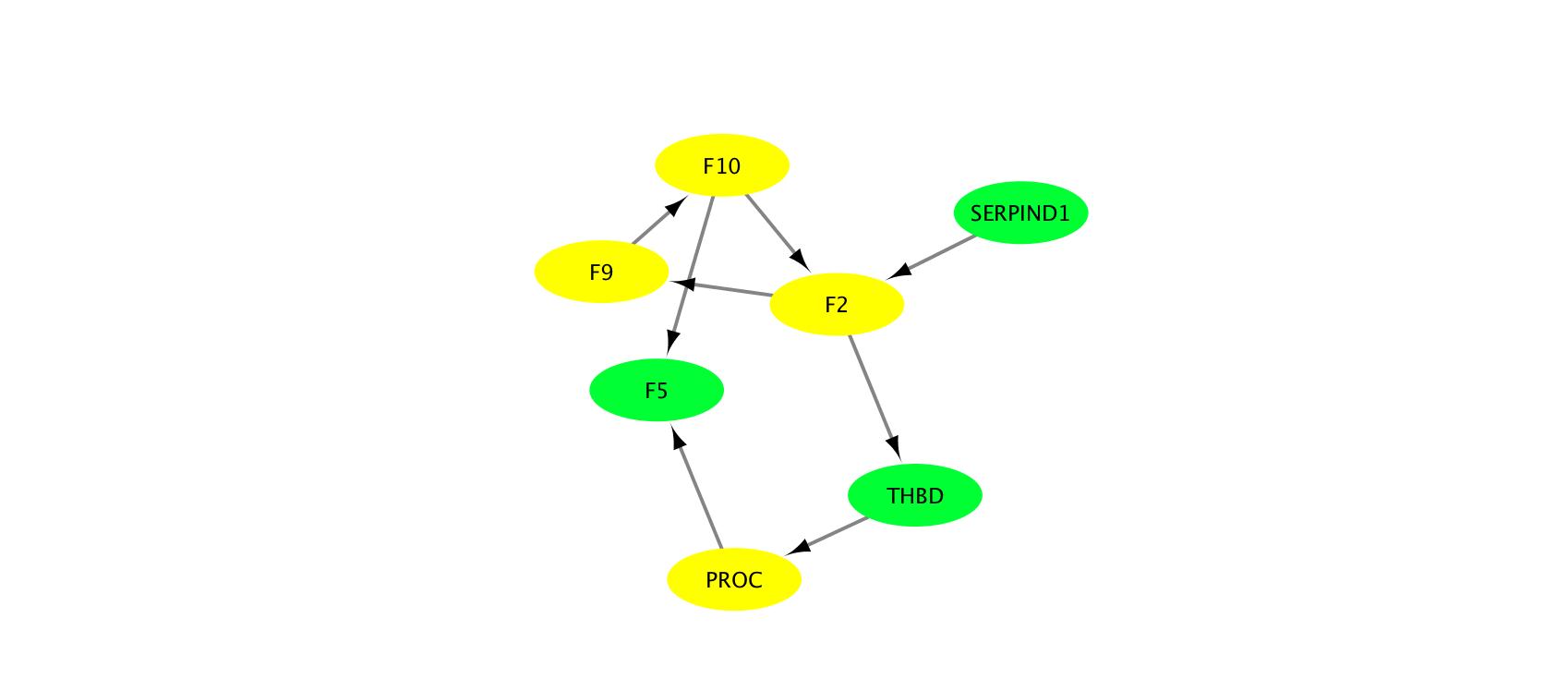


**Supplementary Figure 2.** (A) Box-and-whisker-plots showing global methylation levels measured in 769 probes within 31 genes, selected from the perturbed module reported in figure 6, in MZ-CRC-1 cells without (CTR) or with everolimus (EV) and/or 5-aza-2’deoxycytidine (AZA). (B) Density plot showing methylation level distributions of treated and untreated samples. (C) Box-and-whisker-plots showing global methylation levels of NGFR, MAPK10, TP53, BAX and BCL2 genes in treated and untreated samples.


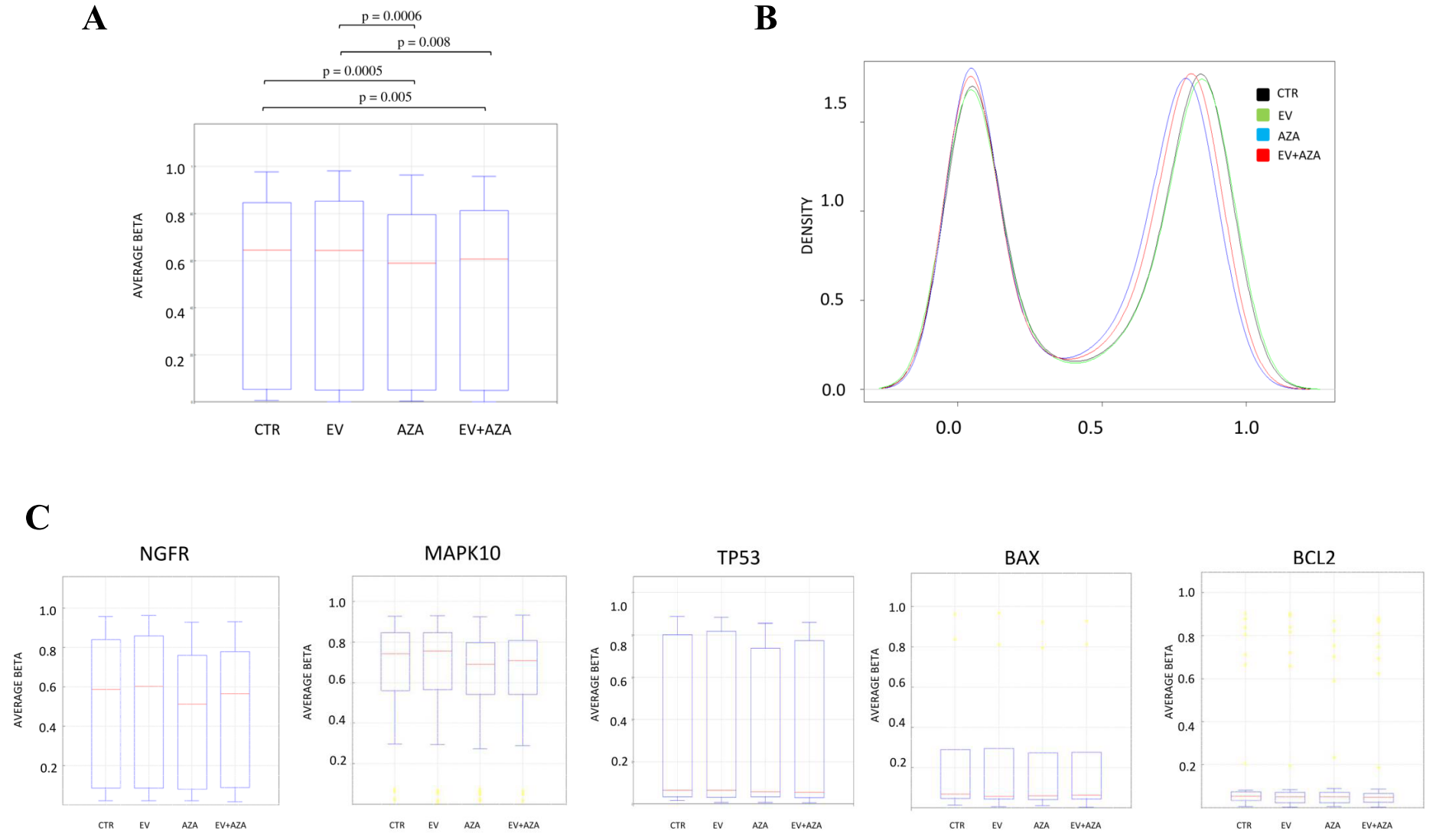

Supplement: Supplementary file 1 — Fig. S1. Perturbed pathway module in the group treated with everolimus. Fig. S2. (A) Box‐and‐whisker‐plots showing global methylation levels measured in 769 probes within 31 genes, selected from the perturbed module reported in Figure 6, in MZ‐CRC‐1 cells without (CTR) or with everolimus (EV) and/or 5‐aza‐2′deoxycytidine (AZA). (B) Density plot showing methylation level distributions of treated and untreated samples. (C) Box‐and‐whisker‐plots showing global methylation levels of NGFR, MAPK10, TP53, BAX and BCL2 genes in treated and untreated samples. Table S1. Differentially expressed genes (DEGs) following incubation with everolimus vs untreated control identified by Significance Analysis of Microarray (SAM), using a delta value of 0.46. Fold change (FC). Table S2. Differentially expressed genes (DEGs) following incubation with AZA vs untreated control identified by Significance Analysis of Microarray (SAM), using a delta value of 0.102. Fold change (FC). Table S3. Differentially expressed genes (DEGs) following incubation with everolimus plus AZA vs untreated control identified by Significance Analysis of Microarray (SAM), using a delta value of 0.46. Fold change (FC). Table S4. Genes in the perturbed everolimus plus AZA module. The gene ID, the gene symbol and if the gene is DEG (1) or not DEG (0) are reported. Table S5. Genes in the perturbed everolimus module. [file MOL2-11-1007-s001.docx]
